# Precise, Genotype-First Breast Cancer Prevention: Experience With Transferring Monogenic Findings From a Population Biobank to the Clinical Setting

**DOI:** 10.3389/fgene.2022.881100

**Published:** 2022-07-22

**Authors:** Hannes Jürgens, Laura Roht, Liis Leitsalu, Margit Nõukas, Marili Palover, Tiit Nikopensius, Anu Reigo, Mart Kals, Kersti Kallak, Riina Kütner, Kai Budrikas, Saskia Kuusk, Vahur Valvere, Piret Laidre, Kadri Toome, Kadri Rekker, Mikk Tooming, Ülle Murumets, Tiina Kahre, Krista Kruuv-Käo, Katrin Õunap, Peeter Padrik, Andres Metspalu, Tõnu Esko, Krista Fischer, Neeme Tõnisson

**Affiliations:** ^1^ Tartu University Hospital, Clinic of Hematology and Oncology, Tartu, Estonia; ^2^ University of Tartu, Clinic of Hematology and Oncology, Tartu, Estonia; ^3^ Department of Clinical Genetics, Institute of Clinical Medicine, University of Tartu, Tartu, Estonia; ^4^ Genetics and Personalized Medicine Clinic, Tartu University Hospital, Tartu, Estonia; ^5^ Estonian Biobank, Institute of Genomics, University of Tartu, Tartu, Estonia; ^6^ Institute for Molecular Medicine Finland, Helsinki Institute of Life Science, University of Helsinki, Helsinki, Finland; ^7^ North-Estonian Medical Center, Oncology and Haematology Clinic, Tallinn, Estonia; ^8^ Institute of Mathematics and Statistics, University of Tartu, Tartu, Estonia; ^9^ Tartu University Hospital, Tartu, Estonia; ^10^ Antegenes, Tartu, Estonia

**Keywords:** genotype-first approach, return of results to biobank participants, research findings/results in healthcare, clinical practice personalized medicine, precision screening

## Abstract

Although hereditary breast cancer screening and management are well accepted and established in clinical settings, these efforts result in the detection of only a fraction of genetic predisposition at the population level. Here, we describe our experience from a national pilot study (2018–2021) in which 180 female participants of Estonian biobank (of >150,000 participants in total) were re-contacted to discuss personalized clinical prevention measures based on their genetic predisposition defined by 11 breast cancer–related genes. Our results show that genetic risk variants are relatively common in the average-risk Estonian population. Seventy-five percent of breast cancer cases in at-risk subjects occurred before the age of 50 years. Only one-third of subjects would have been eligible for clinical screening according to the current criteria. The participants perceived the receipt of genetic risk information as valuable. Fluent cooperation of project teams supported by state-of-art data management, quality control, and secure transfer can enable the integration of research results to everyday medical practice in a highly efficient, timely, and well-accepted manner. The positive experience in this genotype-first breast cancer study confirms the value of using existing basic genomic data from population biobanks for precise prevention.

## Introduction

Pathogenic variants (PV) in known breast cancer (BC)-associated genes have been explored since the identification of the *BRCA1* risk variant in 1994 (Miki et al., 1994). Numerous high- and moderate-risk genes associated with BC and ovarian cancer (OC), and specifically hereditary breast and ovarian cancer (HBOC) syndrome, have been described (Weitzel et al., 2019; Angeli, Salvi and Tedaldi, 2020; Yoshida, 2021). Pathogenic variants in the most prevalent *BRCA1* and *BRCA2* genes (detected in ∼1/400 persons in the general population) dramatically increase the relative risk of BC, particularly among premenopausal women. For example, the cumulative lifetime risk of female BC may be as high as 46–87% for *BRCA1* carriers and 38–84% for *BRCA2* carriers; the cumulative risks of OC development by the age of 80 years in these carriers are 44 and 17%, respectively (Petrucelli, Daly and Pal, 1993). Male carriers of *BRCA1/2* variants are at greater relative risk of developing breast, prostate, pancreatic, and several other malignancies (Kote-Jarai et al., 2011; Moran et al., 2012; Mersch et al., 2015; Mano et al., 2017; Ibrahim et al., 2018; Lee, Lee and Li, 2021; Maccaroni et al., 2021). Pathogenic variants in *TP53*, *STK11*, *PTEN*, and *CDH1* genes are rare but highly penetrant and associated with high risk of developing several different cancers including BC (Chen and Lindblom, 2000; Heitzer et al., 2013; Fusco et al., 2020). Moderate risk genes *ATM*, *PALB2*, *CHEK2*, *NBN*, and *NF1* are associated with 2–5 times higher relative risk for breast and other cancers depending on specific gene (Foretová et al., 2019; Hu et al., 2020; Rainville et al., 2020). Today, clinical genetic testing for the determination of BC susceptibility is widely available in most developed countries. However, guidelines restrict such testing to those who meet certain criteria or thresholds for risk variant carriage likelihood based on personal and family backgrounds of cancer, especially at a young age (National Institute for Health and Care Excellence, 2013; NCCN, 2021). Thus, genetic testing is often performed too late, when several cancers have already been diagnosed in individuals’ families and effective, life-saving preventive measures cannot be applied with full potential (King, 2001; Rebbeck et al., 2004; Rebbeck, Kauff and Domchek, 2009; Singer, 2021). In addition, the application of family history (FH)-based testing criteria is known to result in the identification of less than half of disease-causing variant carriers (Møller et al., 2007; Metcalfe et al., 2010; Gabai-Kapara et al., 2014; Manchanda et al., 2015) After more than 25 years of research and knowledge accumulation, along with tremendous progress in technology and biobank development, systematically informed genetic testing for the detection of fatal cancer susceptibility genes is still not taking place in a population-based manner. Estimates suggest that the implementation of such testing in the United Kingdom and United States alone would prevent hundreds of thousands of cancer cases and save lives cost-effectively (Manchanda et al., 2018; Manchanda and Gaba, 2018). In contrast to current practices, a genotype-first approach is needed to reach this goal; the advantages and challenges of such an approach have been described in previous work from the Estonian Biobank (EstBB) (Leitsalu et al., 2021). In this study, we evaluated the genotype-first approach for precise BC prevention in the clinical setting, assessing its feasibility and acceptance by participants. Breast cancer incidence in clinical and family-based cohorts may differ from population cohorts, therefore we added a cumulative incidence analysis to check the justification of genetics-based intervention approach.

We also propose a service model for early cancer screening that includes genetic testing for application at the national healthcare level.

## Materials and Methods

**FIGURE 1:**
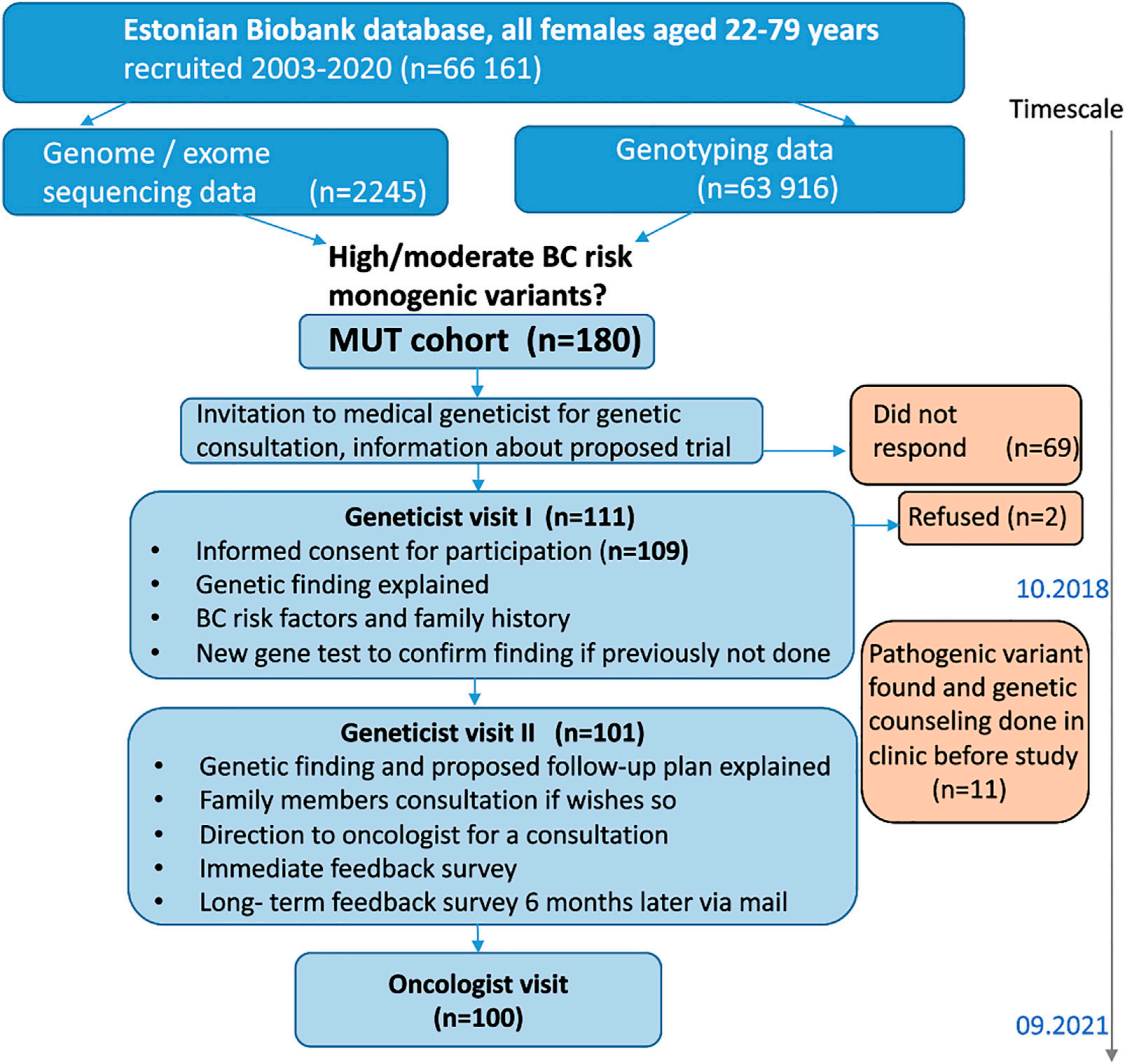
Study design.

This study was designed and performed as an observational feasibility study with clinical implications (Figure 1).

### Ethical Approval

The protocol (and further amendments) for this study was approved by the Ethics Review Committee on Human Research of the University of Tartu and Estonian Committee on Bioethics and Human Research (approvals No 282/T-29, 291/M-21, 1.1-12/643, 11.1-12/643, 1.1-12/1,064, 1.1-12/572). The trial has been registered at ClinicalTrials.gov (no. NCT03989258).

### Study Cohort

The EstBB is a population-based biobank at the Institute of Genomics, University of Tartu, Estonia. It has now over 200,000 participants, representing about 20% of Estonia’s adult population. All participants have provided broad written consent, allowing the EstBB to follow their cases through linked electronic health records from national registries and to re-contact them (Leitsalu et al., 2021). We used EstBB data to confirm and recontact 180 EstBB female participants aged 22–79-years with monogenic variants conferring BC risk in any of the 11 genes listed in relevant clinical guidelines (National Comprehensive Cancer Network, 2017). High-risk genes were *BRCA1*, *BRCA2*, *TP53*, *STK11*, *PTEN*, and *CDH1*, and moderate-risk genes were *ATM*, *PALB2*, *CHEK2*, *NBN*, and *NF1*. BC-associated variants for return of results were identified directly from high-coverage (30×) genome sequencing (*n* = 2,420) and exome sequencing (*n* = 2,356). Next generation sequencing (NGS) data included geographically distributed EstBB participants (*n* = 4,776), of whom 2,245 were female (47%). In addition, we used a subset of array genotyped (Global Screening Array; Illumina Inc. United States) and imputed data available prior to the return of results study (*n* = 154,201) of whom 100,731 were female participants (65.32%).

To analyze the cumulative incidence of BC, we used combined NGS and array genotyped data available from the full cohort for 136,043 EstBB female participants of whom 449 were with confirmed BRCA1 (*n* = 153), BRCA2 (*n* = 92) and CHEK2 (*n* = 204) pathogenic and likely pathogenic variants. All female participants in the EstBB cohort were included in the analysis of BC risk and cumulative incidence. In case of confirmed diagnosis of BC, participants for whom the date of diagnosis was known, were included.

### Variant Detection and Evaluation

The genome and exome sequencing data preparation and quality control workflow has been described elsewhere (Leitsalu et al., 2021). Genotypes from sequenced data were called by GATK (McKenna et al., 2010; Auwera et al., 2013) HaplotypeCaller algorithm. Variants were filtered by GATK Variant Quality Score@ Recalibration (VQSR), call rate <90%, and Hardy-Weinberg equilibrium (HWE) *p* value < 1e-9. The genotype calling for the microarrays was performed by Illumina’s GenomeStudio v2.0.4 software using Illumina Global Screening Array (GSA) v1.0, v2.0, v2.0_EST and v3.0_est arrays. Individuals were excluded from the analysis if their call-rate was <95% or sex defined based on heterozygosity of X chromosome did not match sex in phenotype data. Variants were filtered by call-rate < 95% and HWE *p* value < 1e-4 (autosomal variants only). Indirect identification was based on long-range phasing methods (Leitsalu et al., 2021). In addition, an Estonian population-specific reference dataset for high-coverage genome sequencing samples (*n* = 2,297) was used to impute genotypes for 154,201 EstBB participants genotyped with GSA arrays. Haplotype phasing was performed using Eagle software (ver. 2.3) and imputation was performed using Beagle software (ver. 5.1) (Browning and Browning, 2007; Loh et al., 2016; Mitt et al., 2017). Human reference genome assembly GRCh37 (hg19) was used for all variant analyses.

**FIGURE 2:**
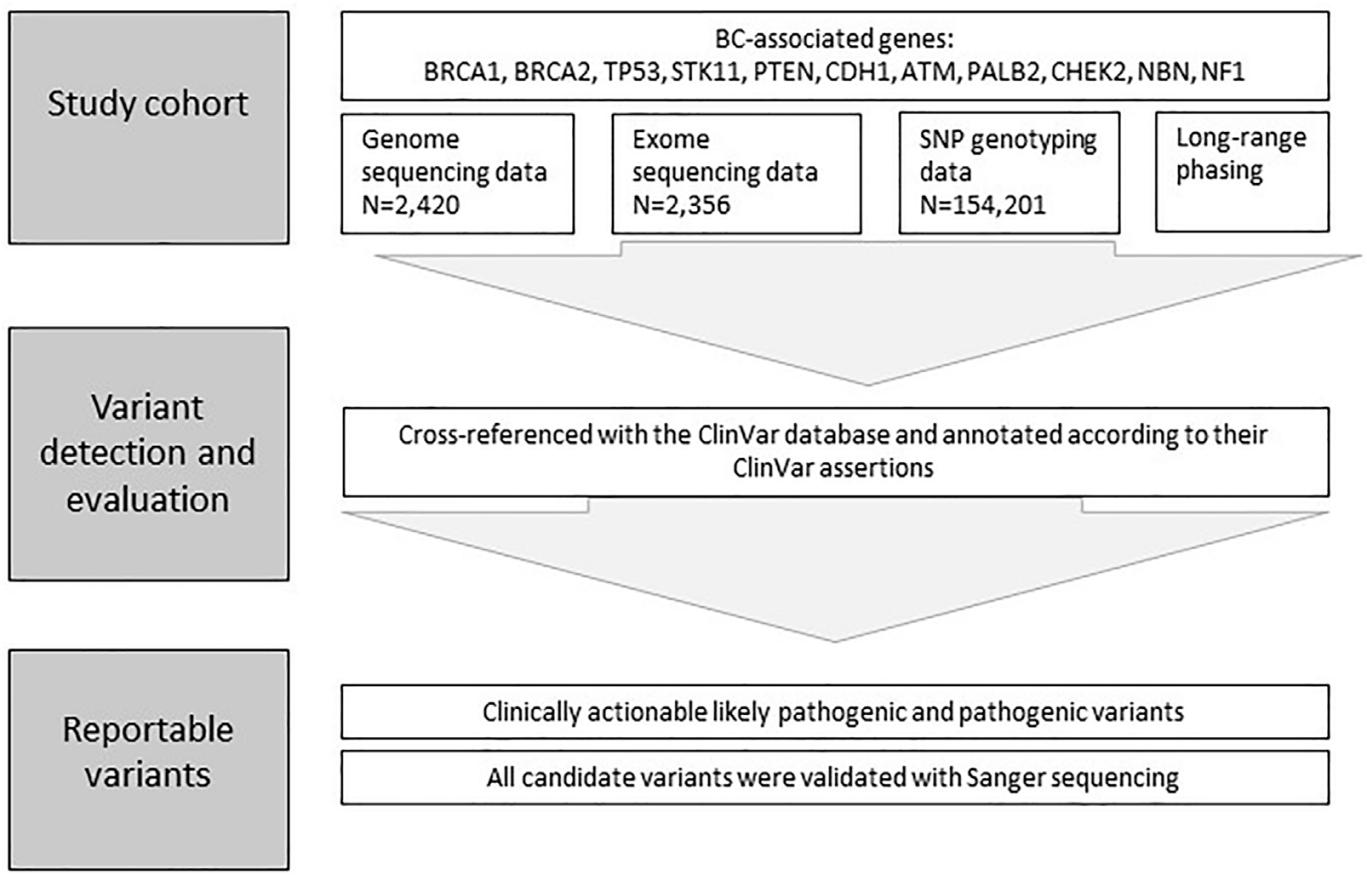
Framework for Variant detection and evaluation. A custom pipeline was used to annotate and prioritize variants in all of the 11 BC-associated genes from sequencing, SNP genotyping and long-range phasing data.

A custom pipeline was used to annotate variants in all of the 11 BC-associated genes from sequencing, genotyping and imputed data (Leitsalu et al., 2021). All identified BC-associated variants in EstBB female participants were cross-referenced with the ClinVar database and annotated according to their ClinVar assertions as pathogenic, likely pathogenic, uncertain significance, benign, likely benign, or with conflicting interpretations of pathogenicity. Identified BC-associated variants were then classified as moderate or high risk based on annotated data if their clinical significance was reported to be likely pathogenic and pathogenic, respectively, according to ClinVar database. This set of identified variants were further prioritized and selected for validation, if carrier status was present in both, directly genotyped and imputed dataset and located in high-risk BC genes, e.g., *BRCA1*/*2.* Also, a subset of high and moderate risk variants in *BRCA1*, *BRCA2*, *CHEK2*, *ATM*, *NBN*, *NF1* from sequencing data were selected for validation. All candidate variants were confirmed with Sanger sequencing prior to participant re-contact using DNA samples stored in the EstBB (Figure 2).

### Recruitment

We identified and re-contacted female EstBB participants with alleles known to confer high or moderate hereditary BC risk. These individuals were sent invitation letters containing brief information about the scientific project on BC, study visits, and results dissemination (i.e., the opportunity to get personal BC prevention plans). To receive further information and to participate, the individuals were asked to schedule initial visits with clinical geneticists. The invitation contained no information on individuals’ personal genetic risks. One month later, a repeat invitation letter was sent to invited individuals who had not scheduled visits. Another month later, study personnel contacted non-responders by telephone.

### Study Actions

The visits were scheduled in the two largest Estonian regional hospitals: Tartu University Hospital and The North Estonia Medical Center. During the first visits, clinical geneticists informed individuals about the study and enrolled only those who consented to receive genetic information. New blood samples were obtained for the second validation with Sanger sequencing, and data on 13 different BC risk factors (American Society of Clinical Oncology (ASCO), 2014; Barnard, Boeke and Tamimi, 2015; Jones et al., 2017; Collaborative Group on Hormonal Factors in Breast Cancer et al., 2019; Zeinomar and Knight, 2019; Zeinomar and Phillips*.*, 2019), cancer FHs, and family pedigrees were recorded.

During the second visits, the geneticists communicated confirmed genetic findings to participants and introduced relevant recommendations from local familial BC prevention and early detection guidelines (based on international guidelines) (National Institute for Health and Care Excellence, 2013; Paluch-Shimon et al., 2016; National Comprehensive Cancer Network, 2017; European Reference Networks and ERN, 2021; NCCN, 2021). Biological relatives of individuals with high genetic risk were mapped and invited for genetic counseling and testing, as part of routine clinical genetic management.

For clinical activities, the geneticists directed participants to clinical oncologists (*n* = 4). These oncologists identified other known BC risk factors, performed clinical examinations, organized imaging studies and laboratory testing (i.e., for the serum OC markers cancer antigen 125 and human epididymis secretory protein 4), discussed additional preventive options (e.g., surgical and chemoprevention), and created personal (variant-specific) surveillance plans according to local guidelines for familial BC management (based on international guidelines) (National Institute for Health and Care Excellence, 2013; Paluch-Shimon et al., 2016; National Comprehensive Cancer Network, 2017; European Reference Networks and ERN, 2021; NCCN, 2021). All previous cancer diagnoses were confirmed with information from digital health records.

### Participant Feedback

Participants’ responses to the receipt of genetic risk information were gathered using two surveys developed based on findings from analogous previous studies (Marteau and Bekker, 1992; Brehaut et al., 2003; Gray et al., 2014; Leitsalu et al., 2016, 2021). The first survey, filled out at the end of the second clinical geneticist visit (immediately after the receipt of genetic risk information) included questions about participants’ satisfaction and solicited self-reported psychological responses to the information received. The second survey, administered ≥6 months later, included questions about decision regret, perceived personal control and coping, psychological adjustment, communication, support, and reported health behavior and healthcare utilization.

### Data Analysis

We used the RedCap database (Harris et al., 2009, 2019) hosted by University of Tartu for information storage and basic descriptive statistical analysis. Detailed analysis was performed with R version 4.0.4 or later (R Foundation for Statistical Computing, 2021). We used the genetic testing criteria from the 2018 and 2021 National Comprehensive Cancer Network guidelines (National Comprehensive Cancer Network, 2017; NCCN, 2021) as clinical practice genetic testing eligibility criteria to evaluate participants and their family history to be indicative for genetic testing. We adjusted testing criteria secondarily to investigate if it would have improved genetic testing detection rate. Adjustment consisted of inclusion all pancreatic and prostate cancer in family history and later (up to age 60) BC onset age for close relatives.

The methodology for time to event data analysis (implemented in R package survival) (Terry M Therneau, 2022) was used to analyze cumulative incidence of BC, with Kaplan-Meier method used to obtain the cumulative incidence curves and Cox proportional hazards model was used to obtain the corresponding hazard ratios with 95% confidence Intervals. EstBB performs regular updating of participants’ health data from the national E-Health database. First time breast cancer diagnosis C50 ICD-10 entry in Estonian national E-health database was used for the analysis.

## Results

### Participation

Of 180 EstBB participants invited to the study, 111 (62%) women scheduled initial visits and 109 (61% of invited, 98% of responders) provided informed consent to study participation. Of these 109 participants, 101 (93%) attended second visits and 100 (92%) met oncologists during the study period.

### Findings

The mean age of the participants at the time of study entry was 48 (range, 28–80) years (<40 years, 32%; 40–49 years, 28%; ≥60 years, 23%). The monogenic BC variant distribution among counseled participants was as follows: 54.1% *BRCA1*, 23% *BRCA2,* 14.7% *CHEK2*, 5.5% *ATM*, 1.8% *NBN*, and 0.9% *NF1*. Risk-conferring variants were pathogenic in 95% of cases and likely pathogenic in 5% of cases (Table 1).

**TABLE 1:** Details of identified pathogenic and likely pathogenic variants.

Gene	rs ID	RefSeq	CDS position	Protein change	Cases per gene (%)	ClinVar
*BRCA1*	rs80357906	NM_007,300	c.5329dupC	p.Gln1756Profs*74	31 (52.5)	KP
*BRCA1*	rs80357711	NM_007,300	c.4035delA	p.Glu1346Lysfs*2	23 (39)	KP
*BRCA1*	rs80357282	NM_007,300	c.1840A > T	p.Lys614*	4 (6.8)	KP
*BRCA1*	rs80357305	NM_007,300	c.4258C > T	p.Gln1420*	1 (1.7)	KP
*BRCA2*	rs80359112	NM_000,059	c.8572C > T	p.Gln2858*	22 (88)	KP
*BRCA2*	rs886040543	NM_000,059	c.467_468insT	p.Lys157fs*26	2 (8)	KP
*BRCA2*	rs1555288494	NM_000,059	c.9097_9098insT	p.Thr3033Ilefs*11	1 (4)	KP
*CHEK2*	rs555607708	NM_007,194	c.1100delC	p.Thr367Metfs*15	9 (60)	KP
*CHEK2*	rs587782401	NM_007,194	c.319+2T > A	NA	4 (26.7)	LP
*CHEK2*	rs121908698	NM_007,194	c.444+1G > A	NA	2 (13.3)	KP
*ATM*	rs587782652	NM_000,051	c.8147T > C	p.Val2716Ala	1 (16.7)	KP
*ATM*	rs780905851	NM_000,051	c.8565T > G	p.Ser2855Arg	1 (16.7)	LP
*ATM*	rs758081262	NM_000,051	c.2554C > T	p.Gln852*	2 (33.3)	KP
*ATM*	rs730881336	NM_000,051	c.742C > T	p.Arg248*	2 (33.3)	KP
*NBN*	rs587776650	NM_002,485	c.657_661delACAAA	p.Lys219Asnfs*16	2 (100)	KP
*NF1*	rs772295894	NM_000,267	c.6792C > G	p.Tyr2264*	1 (100)	KP

NA, not applicable; CDS, coding sequence; KP, known pathogenic; LP, likely pathogenic.

### Previous Genetic Counselling

Eleven (10%) participants had received formal hereditary BC diagnoses and counseling within the medical healthcare system prior to our study. Genetic testing had been performed previously due to personal histories of cancer in three of them. The majority of these participants (*n* = 8) did not request additional geneticist and/or oncologist consultations. Participants’ personal histories contained 16 (15%) cancer diagnoses, most prevalently BC (*n* = 10) and OC/adnexal cancer (*n* = 3; Table 2).

**TABLE 2:** Cancer diagnoses and age of onset.

Age of onset 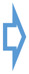	<40 years old	40–49 years old	50–59 years old	>59 years old
Cancer location 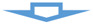				
Salivary gland	0	0	0	1
Skin	0	1	0	0
Breast	3	4	1	2
Ovary/adnex	0	0	2	1
Kidney	0	1	0	0

### Cumulative Breast Cancer Incidence

For *BRCA1* or *BRCA2* variant carriers the hazard of BC is 12.1 (95% CI 9.1–16.0) times higher as compared to the rest of the EstBB cohort. For *CHEK2* variant carriers the hazard is 4.4 (95% CI 2.7–10.7) times higher.

**FIGURE 3:**
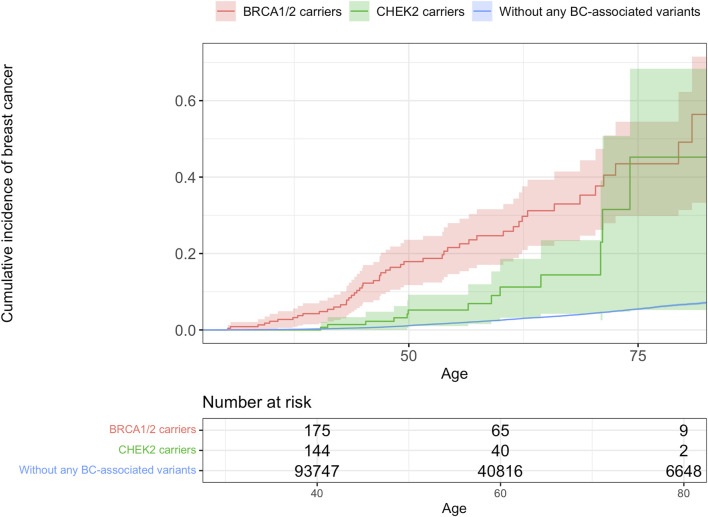
Cumulative incidence of breast cancer.

The cumulative incidence of BC by the age of 70 was estimated to be 35.3% (95% CI 24,7–44.4%) for *BRCA1* or *BRCA2* variant carriers. For *CHEK2* carriers it was estimated to be 14.4% (95% CI 4.2–23.5%) and for the rest of the EstBB cohort without any BC-associated variants it was 4.3% (95% CI 4.2–4.5%) (Figure 3).

### Cancer in Family History

Participants’ FHs revealed cancer among first-degree relatives in 53% of cases and among second-degree relatives in 73% of cases. The most frequent cancer locations were breast (*n* = 77), stomach (*n* = 23), and ovaries (*n* = 16). The remaining 61 cases were primary cancers of seven types (including unknown).

FHs met the 2018 familial BC genetic testing criteria in only 33% (*n* = 36) of cases. With criteria adjustment (including for pancreatic and prostate cancer histories and later BC onset age), another 20% of participants would have met the criteria, meaning that slightly more than half (53%) of actual risk variant carriers would have been identified by FH-based testing.

### Breast Cancer Risk Factors

Information on BC risk factors in the study participants is provided in Table 3. The mean ages of menarche and menopause were 13.3 (range, 10–16) and 49.2 (range, 35–57) years, respectively. The mean numbers of pregnancies and childbirths per participant were 2.55 (range, 0–8) and 1.85 (range, 0–5), respectively; the mean age at the time of first labor was 23.7 (range, 16–40) years. The mean body mass index was 26.4 kg/m^2^. The most prevalent hormonal treatment was estradiol; in 90% of cases, the exact drug was not known. Half (50%) of the participants used oral contraception, for a mean of almost 7 (range, 0.1–20) years in total and 2.2 (range, 0–20) years before the first childbirth. Almost 70% of participants reported at least some alcohol consumption; 52% consumed <1 unit per week (social drinkers), 23% consumed ≥1 unit per week, and no participant reported daily alcohol consumption. 14% of participants were current regular smokers and 16% were former smokers. The mean number of pack-years was 14. Besides genetic findings, 46% of participants had one and 23% had two additional BC risk factors.

**TABLE 3:** Factors increasing the relative risk of invasive breast cancer in the study participants.

Risk factor	Percentage (%)
	of all participants
*BRCA1/BRCA2* risk variant carrier	77.06
Other breast cancer pathogenic gene variant carrier	22.94
Atypical hyperplasia	0.92
Previous biopsy or operation on breast	0.92
First degree relative with breast cancer	21.10
Menopausal hormone therapy containing estrogen and progestin	0.00
Obesity (BMI >30)	23.85
Early menarche (before age 12)	9.17
Giving birth at an older age (after 35 years of age)	1.83
Having no children	14.68
Late menopause (after 55 years of age)	3.67
Menopausal hormone therapy containing estrogen only	0.92
Alcohol consumption (=/>1 unit per day)	0.00
Using hormonal birth control methods (before first childbirth)	9.17
Smoking (regular, incl. former regular smoking)	29.36

### Diagnostic and Physical Examination Results

BC (incident cases) was diagnosed in six participants (5.5%) and in one case it was bilateral. Together with prevalent cases (10), BC was the most frequent (14.7%) malignancy among participants. Physical examination yielded pathological findings in three (3.3%) cases, and the diagnosis of invasive cancer was confirmed in one of these cases. Breast biopsies were performed in five cases. Seventy-three percent of participants underwent digital mammography; findings were pathological in four cases and unclear in two cases.

Breast magnetic resonance imaging and ultrasound examinations were performed for 43 and 6% of participants, respectively. Gynecological examinations and gynecological ultrasound were performed in 27 and 48% of participants, respectively. Serum OC markers were assessed in one-third of cases. Two *CHEK2* variant carriers underwent colonoscopy, which yielded the finding of tubular adenoma in one case. One *BRCA1* carrier with a personal history of BC underwent a positron emission tomography/computed tomography examination, which revealed no evidence of disease. During follow-up period, a stage I kidney cancer was discovered incidentally with ultrasound in a 47-year-old *BRCA1* carrier and later she was successfully operated on.

Information on participants with BC and other cancer diagnoses is provided in Tables 4, 5, respectively.

**TABLE 4:** Prevalent and incident breast cancer cases.

ID	Age at dx	Preva-lent/Inci-dent	Y.o.d	Morphology	Stage	Family history: close blood relative with BC/OC/PC/pac/gc and age of onset	Gene	Risk-reducing surgery
1	32	I	2021	IDC G3, TNBC; + DCIS focal lesions, Ki67-80%	IB	Mother´s side: BC, 38, 46, 47; OC, 56, 57	BRCA1	no
2	34	P	2010	IDC G3, ER+, PR+, HER2-neg. Ki67-20%	IIIA	Mother’s side OC, 44; BC, 42; male BC, 50	BRCA2	yes (BSO 2010)
3	34	P	2007	IDC, ER+, PR+, HER2-neg	IIIA- > IV	Father´s side, unk. primary, older age	BRCA1	no
4	35	P	1986	-	-	Mother’s side gyn. cancer in 70-s; Father’s side 2 unk. cancers in 40-s	BRCA1	no
5	37	I	2020	IDC G3, TNBC; Ki67-50%	IIIC	Mother, gyn. cancer, 40	BRCA1	yes (BSO 2021)
6	40	P	2014	IDC (m3), G3 ER-, PR-, HER2-pos	IIB	Mother’s side 2 unk. lethal malignancies in their 40-s; Father’s side BC, 50-s	CHEK2	no
7	41	I	2021	IDC; cT2N0M0; ER+, PR +; HER2-neg; Ki67–27%	IIA	Father´s side PC, 88; GC, 88	BRCA2	yes (BSO + MT 2021)
8	43	P	2016	-	-	Mother BC, 52 and OC, 60-s; mothers mother BC?	BRCA1	yes (BSO + MT 2017)
9	44	I	2019	Left breast: IDC, TNBC, Ki67- > 20%. Right breast: IDC G1 ER+, PR+, HER2-neg, Ki67- < 10%	IA x2	Mother´s side PC, 56	BRCA1	yes (BSO 2019)
10	45	P	2013	-	-	Father´s side BC, 45; OC 50	BRCA1	no
11	47	P	2010	-	-	Mother´s side: BC, 37 + 62 contralateral, 50; OC, 55, 60, 60, 80	BRCA1	yes (BSO + MT 2011)
12	49	I	2021	IDC (m2) G1 + DCIS (multiple); Ki67–23%; ER+, PR+, HER2-neg	I, 0	Mother´s side BC, 44, 75 (mother and grandmother)	BRCA1	yes (BSO + MT 2021)
13	54	P	2014	IDC G3, TNBC	IA	Mother´s side GC, 64	BRCA1	no
14	59	I	2021	IDC G3, TNBC	IA	Mother´s side 3-cases of OC (2 at age 65, 1 NK)	BRCA1	yes (BSO 2021)
15	60	P	2007	ILC G3, ER+, PR+, HER2-neg. Ki67-30%	IA	Mother´s side BC, 80-s	BRCA1	no
16	62	P	2014	DCIS, Ki67-5%, ER+, PR+, HER2-neg	0	Father, GC, 40	BRCA1	no

BC, breast cancer; OC, ovarian cancer; PC, prostate cancer; PAC, pancreatic cancer; GC, gastric cancer; IDC, invasive ductal carcinoma; G3, grade 3; TNBC, triple-negative breast cancer; DCIS, ductal carcinoma *in situ*; Ki67, cellular marker of proliferation; ER, estrogen receptor; PR, progesterone receptor; HER2, human epidermal growth factor receptor two; BSO, bilateral salpingo-oophorectomy; MT, mastectomy; unk., unknown; gyn., gynecological; Y.o.d, year of diagnosis.

**TABLE 5:** Cancers other than BC.

ID	Type of cancer	Age at dx	Preva-lent/Inci-dent	Y.o.d	Morphology	Stage	Family history: close blood relative with BC/OC/PC/pac/gc and age of onset	Gene	Risk-reducing surgery
1	Ovarian	47	P	2004	-	-	Mother’s side BC, 59, 35	BRCA1	no
2	Ovarian	60	P	2018	Serous carcinoma in both ovaries	IIIC	Father’s side GC, 61	BRCA1	no
3	Fallo-pian tube	51	P	2002	G3	IIIC	Sister BC, 45; Father´s side 1 unknown primary cancer	BRCA1	no
4	Kidney	47	I	2020	Chromophobe renal cell carcinoma	IA	Mother’s side: GC, 40s; Father’s side: PC, 61; BC, 80; PAC, 85	BRCA1	yes, (BSO 2021)
5	Kidney	46	P	2000	-	-	no	BRCA1	yes (BSO, 2020)
6	Salivary gland	60	P	2012	Adenocystic carcinoma; Ki67 20%	I	Mother BC, 45	CHEK2	no
7	Skin	43	P	2009	Basalioma	I	Father’s side GC, over 50	BRCA1	no

BC, breast cancer; OC, ovarian cancer; PC, prostate cancer; PAC, pancreatic cancer; GC, gastric cancer; BSO, bilateral salpingo-oophorectomy

### Risk Reduction Interventions

Sixteen (14.7%) prophylactic salpingo-oophorectomies and 5 (4.6%) mastectomies were performed during the study period; no chemoprevention was performed, except for patients with BC who received adjuvant treatment. The majority (62.5%) of prophylactic salpingo-oophorectomies were performed on participants with personal histories of cancer, and 80% of mastectomies involved the removal of contralateral breast tissue in patients with personal histories of BC. FHs were indicative (e.g., fulfilled the testing criteria) for risk variants in 12 (75%) cases, and *BRCA1* or *BRCA2* variants were found in 100% of patients undergoing surgical intervention.

### Clinical Geneticist Activities With Family Members

The scheduling of the first and second visits with clinical geneticists were partially affected by the COVID-19 pandemic. The close relatives of 106 participants were invited for visits, according to the clinical best practice guidelines. The mean number of relatives recommended for counseling per participant was 4.3. In total, 453 invitations for cascade screening were made. The complete number of relatives actually counseled by geneticists is not known.

### Participant Feedback

Response rates for the first and second surveys were 84.4% (*n* = 92) and 47.7% (*n* = 52), respectively. Not all participants responded to all survey questions.

**FIGURE 4:**
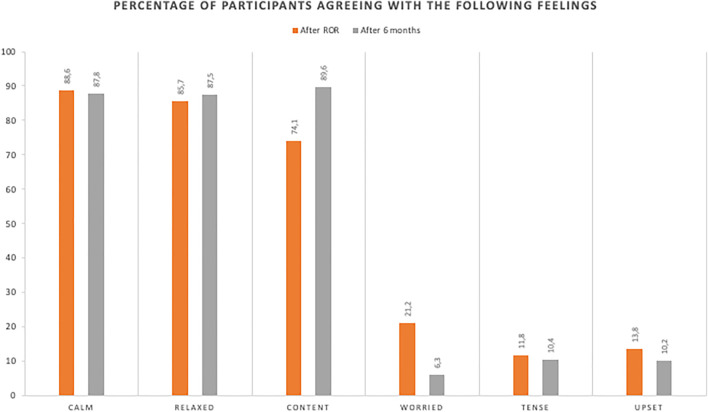
Proportions of participants reporting positive and uncomfortable feelings immediately after receiving genetic risk information and 6 months later.

After receiving their genetic risk information, the majority (74–88%) of respondents tended to feel calm, content, and relaxed; a minority (11–21%) reported feeling worried, upset, or tense (Figure 4). Most respondents considered the information received to be informative (98.8%), valuable (97.6%), understandable (96.7%), and interesting (93.1%). Almost all (97.7%) respondents appreciated being contacted. Similarly, 97.7% of respondents reported understanding the familial implications of the findings and 82.5% thought that they would be able to explain the meaning of the findings to their relatives. Most (95.5%) respondents reported that they knew who to turn to for further information and support, and 97.8% thought that they had received sufficient information through consultation and counseling.

Six or more months later, a greater proportion (87–89%) of respondents tended to feel content and relaxed, and fewer (6–10%) reported feeling worried, upset, or tense.

Most participants (88.2% of 51 individuals) reported that they were coping with having genetic risk factors; one participant disagreed slightly and five (9.8%) were unsure. The majority of respondents considered their decision to participate in the project and receive genetic risk information to be right (96%) and wise (94%), and 92% indicated that they would make the same decision again. The majority of respondents did not consider this decision to have caused them harm (92.1%) and had no regrets (90%). One person regretted the decision. Most (93.9%) of 49 respondents planned to follow the recommended screening and risk management plans.

The participants made some suggestions for improvement. Their comments addressed risk communication “Disclosure of the findings could have been more gentle/considerate”, “The doctor was too abrupt and did not explain much other than the need to hurry and remove my breast and ovaries: (My doctor got me scared so now I have not returned to the doctor for almost a year”), long-term risk management (“More thought on what a person could do moving on, psychologist? Counseling regarding the mastectomy?”) and help with family communication (“Collaboration with family physicians and disclosure to family members through family physicians”).

## Discussion

The results of this study support the genotype-first approach to BC screening. It is applicable and feasible in the clinical setting and could thus be integrated into personalized population-based BC screening programs to maximize the benefits of prevention and early detection strategies. During our study, BC was diagnosed in six participants that would have otherwise been missed or discovered late. Of these six patients, five were younger than the current national screening program entry age of 50 years; two of these cases were diagnosed during pregnancy before the age of 40 years (the recommended age for baseline mammography). Most variant carriers had not been tested previously or were not aware of their genetic status. Furthermore, FHs did not fulfill the criteria for genetic testing in more than half of the cases. This might be partially explained by limited knowledge on the family history of the participants, but also a lower than expected cumulative incidence in the population cohort. Cumulative risk analyses were therefor performed on EstBB samples, genetic and breast cancer data. In our population cohort, *BRCA1*, and *BRCA2* pathogenic and likely pathogenic variants have high risk of breast cancer. *CHEK2* average cumulative breast cancer risks were even higher than expected from literature, but have large confidence intervals (Vahteristo et al., 2002; Weischer et al., 2007, 2008). Despite the relatively small number of subjects, our findings strongly justify the return of results and clinical interventions. The results are also in line with previously published prospective studies on larger cohorts (Mavaddat et al., 2013; Karoline B and Kuchenbaecker K. B. et al., 2017).

### Modifiable Breast Cancer Risk Factors

The most common modifiable risk factor for BC in this study was smoking (current and former), and the second common factor was obesity (BMI>30 kg/m^2^). According to a recent national health report, the average prevalence of smoking among Estonian females is slightly higher (38%) and that of obesity is slightly lower (21%) than in our study group (Reile and Veideman, 2021). In Europe, only 16% of females are obese (Janssen, Bardoutsos and Vidra, 2020; Eurostat, 2021). Obesity is a serious public health problem associated with the development of several chronic diseases; it is responsible for substantial and increasing direct and indirect healthcare costs as it becomes more common in Europe, globally (its prevalence has almost tripled since 1975), and at younger ages (Eurostat, 2021). However, associations of obesity, smoking, and other risk factors with BC have not been found to be sufficiently strong to warrant the recommendation of sole preventive measures for high-risk allele carriers (King, Marks and Mandell, 2003; Manders et al., 2011; Zeinomar and Knight, 2019; Iyengar et al., 2021). Nevertheless, the provision of scientific information on risk factors during consultations with very young high-risk individuals is important to emphasize what they can do on a personal level to improve their health and minimize the risk of developing cancer before the age at which surgery, the most effective but psychologically most difficult preventive measure, is recommended. A review demonstrated that parity (after four births) and breastfeeding protect against BC and OC in *BRCA1* carriers, whereas the opposite is true for *BRCA2* carriers, whose risk of BC increases with each birth and for whom breastfeeding has no protective effect (Fishman, 2010).

Oral contraceptive use has also been associated with a reduced risk of OC development, but a potential slightly increased risk of BC development (Iodice et al., 2010). In this study, we identified BC during pregnancy in two *BRCA1* carriers with the single additional BC risk factor of hormonal birth control use besides their genetic status. Regarding obesity, studies conducted with unselected populations have shown the opposite effect as those conducted with *BRCA1/2* carriers; pre-menopause obesity protects against BC, whereas post-menopause obesity is a risk factor (Karoline B Kuchenbaecker K. B. et al., 2017; Lammert et al., 2018; Manchanda and Menon, 2018; Gallagher et al., 2020; Gao et al., 2021). In this study, we measured the body mass index but did not evaluate diet or physical activity. Data collection on modifiable risk factors was included to enable a longer prospective study on the given cohort and their further inclusion in the complex risk models.

### FHs, Guideline Modification, and New Information

We found that a cancer FH, based on suggested testing criteria, is not indicative of the presence of known genetic disease-causing variants in most cases (National Comprehensive Cancer Network, 2017). We modified the criteria to include FHs in close relatives of prostate, pancreatic, and gastric cancers and BC with an onset age of <60 years, which resulted in the detection of slightly more than half of variants. The use of FHs has several limitations, especially in modern societies characterized by individualism, the privacy of health information, mental and/or physical separation of families, and non-biologically related families, which result in the lack of detailed information on family cancers (e.g., age of onset, anatomical location, morphological form, and immunohistochemical properties such as estrogen and progesterone receptor and human epidermal growth factor receptor two status in breast cancer). The retrieval of detailed and complex FHs can be time consuming and still result in imprecise risk assessment and testing decisions. For these reasons, genetic testing would ideally not be restricted to FH-based criteria, but rather be used to aid the assessment of malignancy prevalence likelihoods in variant carriers for the implementation of preventive strategies (Manchanda and Menon, 2018). Another powerful recently developed tool for risk assessment is the polygenic risk score, used in combination with monogenic testing (Karoline B Kuchenbaecker KB. et al., 2017; Gallagher et al., 2020; Gao et al., 2021; Shah, 2021). Zhang et al. (Zhang et al., 2019) reported that population-level genomic screening of all young adults was applicable and cost effective. Guzauskas et al. (Guzauskas et al., 2020) found that a model of genetic screening for HBOC, including cascade influences, in the United States was moderately cost effective when applied to younger (aged 25–30 years), but not older (≥45 years), individuals.

A systematic review revealed that the current evidence, technology, and knowledge support the application of population-level genetic testing for preventive strategy implementation in the near future (Manchanda and Gaba, 2018). Some issues remain to be resolved, including the development, implementation, and thorough evaluation of alternative service-delivery models that involve genetic experts and downstream management pathways. Manchanda and Gaba (Manchanda and Gaba, 2018) proposed six factors to be considered to maximize the effects of such programs: clinical utility, equal access, widening research, robust implementation pathways, cost effectiveness, and consistent coherent messaging. Supported by our findings and literature we suggest hereditary breast cancer risk screening with genetic testing for the whole population of women aged 25–30 and it should be developed, prepared and implemented wise as soon as possible to save more young lives. Moreover, whenever basic genomic information is available, as is the case with biobanks, it should be searched for hereditary cancer predisposing variants, and systematic return of data activities should be planned.

### Surgical Procedures

During the limited follow-up period of this study, fewer participants than expected decided to undergo risk-reducing surgeries, the most effective preventive measures. This finding is in line with the previously reported 31% risk-reducing salpingo-oophorectomy uptake and no uptake of prophylactic mastectomy during follow-up among EstBB *BRCA1/2* carriers (Leitsalu et al., 2021). It can be explained by the short follow-up period; cultural factors and traditions; the lack of availability of and reimbursement for high-quality breast reconstruction, as well as the inability to consult doctors about such procedures; and several other factors (Scheepens et al., 2021). We did not directly examine these possible explanations for low preventive surgery uptake in this trial. In a prospective study, Chai et al. (Chai et al., 2014)observed risk-reducing salpingo-oophorectomy uptakes of 45% among *BRCA1* carriers and 34% among *BRCA2* carriers by the age of 40 years; these percentages were 86 and 71%, respectively, by the age of 50 years. Risk-reducing mastectomy uptake was estimated to be 46% by the age of 70 years in both groups-(Chai et al., 2014). In our study, 32% of participants were aged <40 years and 60% were aged <50 years.

### Cancers

The cancer burden is going to be a major problem in the next 20 years. Global Cancer Statistics estimates that a 47% increase will occur, with 28.4 million new cancer cases (excluding basal cell carcinoma) diagnosed globally in 2040 (Sung et al., 2021).

Almost 15% of participants and 21% of first-degree relatives in this study had BC diagnoses; one third of diagnoses were made before the age of 40 years, almost half were made at 40–50 years, and only quarter were made in alignment with the current BC screening program age. The estimated prevalence of *BRCA* risk variants in Estonia is 0.8% (1/124), according to EstBB sequencing data (Leitsalu et al., 2021) but it may be as high as 1/40 among Ashkenazi Jews (Hartge et al., 1999). Simple calculations suggest that the adult population of Estonia contains about 75,000 male and female monogenic variant carriers at risk of developing highly aggressive cancers of which many could be prevented or timely discovered and cured with appropriate personalized screening and handling programs following genetic testing. This study, conducted with only female carriers, confirmed that the *BRCA1*-associated BC pathological features in this population are typical [triple (estrogen receptor, progesterone receptor, and human epidermal growth factor receptor 2) negative, high grade, and poorly differentiated in the majority of cases], and that *BRCA2*-induced BC features reflect those of the general BC population more (Mavaddat et al., 2012).

In our cohort we found two kidney cancer cases (1 prevalent and one incident), both with the same *BRCA1* PV (rs ID rs80357711) and early onset (before age 50) which is much different (>50 times higher) from the expected average population levels of kindney cancer prevalence, suggesting a possible connection with specific PV of *BRCA1* and kidney cancer (chromophobe subtype). However, a recent large (with almost 15,000 *BRCA1/2* PV carriers) analysis (Li et al., 2022) did not detect any significant association between kidney cancer and *BRCA1/2* variants leaving little room for our data from small numbers of cases and controls to claim the opposite. Otherwise, our data on *BRCA*1/2 associated cancers beside BC is in line with Li et al. as the second most common cancer (after BC) among first-degree relatives in this study was gastric cancer (Maccaroni et al., 2021; Li et al., 2022).

### Moderate and High-Risk Breast Cancer Variant Detection From SNP Genotyping Data

Less than one-quarter of pathogenic genetic variants in our study participants were detected with sequencing data, and most of them were non-*BRCA* findings; almost all *BRCA1/2* findings were discovered from genotype data. As sequencing technology is more expensive, genotyping is currently the most commonly used method for biobank genetic information processing. This situation poses a challenge when attempting to incorporate biobank data into clinical practice, where reliability is extremely important to include and exclude certain pathologies in differential diagnoses. We see that genotyping-based genetic information is sufficiently trustworthy to be made widely available for some personalized medicine applications in clinical practice in the near future. At the same time, not all known high-disease-risk genetic variants are discovered by genotyping only and the high-risk variants found will have to be confirmed by sequencing before returning to participants. The creation of a system or database for genetic information storage and use at the national healthcare system level is underway in Estonia. All genetic information from biobanks could be transferred to such a system upon donors’ request. The broad-scale utility of such approach will be tested in the near future.

### Participant Feedback

Only 10% of study participants had previously received counseling on possible hereditary BC, and slightly more than half of the participants had FHs indicative of hereditary BC. Thus, participants did not necessarily expect to discuss the topic of BC or receive news of the genetic risk factors they had. Nevertheless, they tended to feel calm, relaxed, and content; a minority of participants reported uncomfortable feelings, but almost all participants viewed the information they received as valuable and appreciated being contacted.

Although most participants reportedly planned to follow the screening and risk management recommendations, the uptake of risk-reducing surgeries was relatively low. Furthermore, some participants expressed a preference for more subtle and less direct communication when specialists introduced the idea of such surgery.

### Limitations

This study has a number of minor limitations. The participation rate was lower than expected (61%), which might be related primarily to the biobank setting. The letter presenting only general information about the study may not have been sufficiently specific to be taken seriously, and difficulties with making contact after long periods (for some, almost 20 years later e.g., wrong addresses, loss of interest) were inherent. In addition, the COVID-19 pandemic occurred during the second part of the trial period and probably also impacted the participation rate, intervals between visits to geneticists, referrals from geneticists to oncologists, and oncologist visits (including rates of prophylactic surgery uptake and performance). Another limitation is that the trial participants did not comprise the full set of HBOC-associated gene carriers in the EstBB cohort; they were a budget-limited, randomly selected sub-population, preferably carrying high-risk gene variants. In addition, the clinical information gathered may not have been complete, as private medicine actions and genetic testing information were not available to oncologists and thus may not reflect the real rates reported. However, private oncological medicine has had a marginal role in Estonia, and the data presented are generally representative. Furthermore, we had difficulty following participants’ relatives due to the lack of registry; they were not involved directly in the study and thus not handled systematically. Finally, oncological FHs are often unclear with respect to specific diagnoses and morphological factors addressed in testing guidelines, such as the metastatic status, intraductal/cribriform morphology of prostate cancer, and diffuse nature of gastric cancer.

## Conclusion

This study supports the applicability and feasibility of the genotype-first approach in the clinical setting of HBOC. This approach could be integrated into personalized population-based BC screening programs to maximize the benefits of prevention and early detection strategies.

## Data Availability

Restrictions apply to the datasets: The datasets presented in this article are not readily available because include personal information of subjects. Requests to access the datasets should be directed to Ethics Review Committee.
